# Ten Years of a Trans-Pacific Medical Education Partnership—Training Globally to Serve Locally

**DOI:** 10.31486/toj.23.0081

**Published:** 2023

**Authors:** G. Dodd Denton, Leonardo Seoane, Diann S. Eley

**Affiliations:** ^1^ Department of Internal Medicine, Ochsner Clinic Foundation, New Orleans, LA; ^2^ The University of Queensland Medical School, Ochsner Clinical School, New Orleans, LA; ^3^ Division of Academics, Ochsner Clinic Foundation, New Orleans, LA

**Keywords:** *Education–medical*, *global health*, *international medical school*

## Abstract

**Background:** In 2009, Ochsner Health in New Orleans, Louisiana, and The University of Queensland (UQ) in Brisbane, Queensland, Australia, formed a medical school partnership. The rationale for UQ to enter this partnership was to strengthen its already strong international multicultural environment and enrich the domestic Australian student experience. The rationale for Ochsner Health was to raise its academic stature and to train high-quality physicians. This partnership is unique among US international partnerships because the intent is for graduates to practice in the United States.

**Methods:** A new 10-year agreement began in January 2020 with further enhancements to the program. This article describes the educational philosophy informing the partnership, the programmatic design, challenges faced and overcome, and outcomes from the first 10 graduating cohorts of this medical program.

**Results:** The UQ-Ochsner Clinical School partnership posed many challenges. UQ faced a major cultural shift to implement United States Medical Licensing Examination step preparation. Student recruitment challenges and state-specific accreditation concerns had to be solved. The coronavirus disease 2019 pandemic presented unique challenges with the strict prohibition on travel into Australia. Challenges were addressed, and the tenth graduating class completed training in December 2021. More than 850 medical students have graduated from the program, with 30% staying in Louisiana for postgraduate training. The overall first-attempt match rate of 95% exceeds the US allopathic average. Although graduates have faced stigma from their designation as international medical graduates, they have successfully matched in every specialty and in almost every US state.

**Conclusion:** The UQ-Ochsner Clinical School partnership has been successful for the institutions involved and the students who have graduated. The overarching aim of the partnership, “train globally to serve locally,” has endured. Through their training in this partnership, UQ-Ochsner Clinical School graduates bring a unique global outlook to their roles while helping to fill the increasing need for physicians in the United States.

## INTRODUCTION

Many aspiring physicians today foresee their future careers as not only improving the health of individuals in their own community but also improving health care globally. Training college graduates with these goals in mind can be a challenge for medical training programs. Health care in the United States is a dynamic landscape shifting toward primary care and emphasizing population health while facing a physician shortage of 37,800 to 124,000 physicians by 2034.^1^ The trans-Pacific partnership between The University of Queensland (UQ), Brisbane, Queensland, Australia, and Ochsner Health, New Orleans, Louisiana, is designed to train physicians in a global cross-cultural environment for practice in the United States.

The UQ-Ochsner Clinical School partnership was founded in 2009 to give US citizens and permanent residents who are college graduates the opportunity to obtain a medical degree integrated across 2 continents. Students complete their first 2 years of preclinical medical education in Brisbane, Queensland, in Australia, and their final 2 clinical training years in New Orleans, Louisiana, in the United States. The partnership trains students in 2 different educational, social, and cultural environments and in 2 different health care systems, providing graduates with a broad perspective on health care delivery. Graduates of the program are eligible to enter specialty training in either the United States or Australia. The partnership has proven to be mutually beneficial by leveraging cross-cultural experiences and expanded professional networks to enhance the skill sets of domestic and international UQ students, medical educators, and research collaborators.

In this article, we describe the educational philosophy informing the partnership, the programmatic design, challenges faced and overcome, and outcomes from the first 10 graduating cohorts of this medical program.

## OVERVIEW OF UNITED STATES MEDICAL SCHOOL PARTNERSHIPS WITH OVERSEAS INSTITUTIONS

Although the UQ-Ochsner Clinical School partnership is unique, it is not the first global collaboration between a US medical school and a medical center or university in a foreign country.^2^ The Columbia University College of Physicians and Surgeons in New York and Ben Gurion University of the Negev in Israel have had a collaborative program since 1998. The Medical School for International Health in Israel focuses on cross-cultural and international medical practice and attracts students from all over the world.^3^

In 2001, Cornell University in Ithaca, New York, became the first US university to provide its medical degree in a foreign country. Weill Cornell Medicine-Qatar is a partnership between Cornell University and the Qatar Foundation and is part of Weill Cornell Medicine. Weill Cornell Medicine-Qatar was the first medical school established in Qatar and is a pioneer of coeducation in its university system. Students come from a broad range of countries with the objective of practicing in their home communities.^4^

Another long-established partnership is between Duke University in Durham, North Carolina, and the National University of Singapore (NUS), known as the Duke-NUS Medical School.^5^ Although Singapore already had a medical school in the British tradition (5-year undergraduate-entry program), the desire was to establish a US-style graduate-entry medical program. The agreement between the 2 institutions in 2005 formed the Duke-NUS Graduate Medical School. The curriculum is modeled on the Duke University School of Medicine. Three-quarters of the student cohort are Singaporeans and do their residency training in Singapore.^6^

In 2012, the School of Medicine at the University of Pittsburgh began working with Nazarbayev University in the Republic of Kazakhstan to help establish a medical school based on a US-style curriculum. The first class entered in 2015.^7^

Two consistent features of these partnerships are the perception of the prestige of the partnerships by the institutions involved and the value placed on the global nature of the experiences. The graduates meet the needs of their local communities, and the US partner benefits in a variety of ways. In the UQ-Ochsner Clinical School partnership, graduates practice in the United States after developing a global perspective and having cross-cultural experiences during their training.

## HISTORY OF THE UNIVERSITY OF QUEENSLAND-OCHSNER HEALTH TRANS-PACIFIC PARTNERSHIP

UQ is a public not-for-profit organization founded in 1909. The main campus is in Brisbane, Queensland, along the east central coast of Australia. UQ was globally ranked 51 in the 2021 Academic Ranking of World Universities^8^ and achieves the highest research funding of any Australian university. The UQ medical program was established in 1936 and is the largest in Australia.

Ochsner Health, founded in 1942 in New Orleans, Louisiana, as the Ochsner Clinic by 5 surgeons, has been a training site for medical students from other medical schools in the New Orleans area and has administered its own residency programs since its founding. Ochsner Health is a not-for-profit independent academic health system with more than 37,000 employees and more than 4,700 employed and affiliated physicians in 90+ medical specialties and subspecialties. Ochsner Health operates 46 hospitals and more than 370 health and urgent care centers across Louisiana, Mississippi, and Alabama; has 30 Accreditation Council for Graduate Medical Education–accredited residency programs; and also has programs in translational, clinical, and health services research.^9,10^

At the time the partnership was formed, the UQ School of Medicine offered the bachelor of medicine, bachelor of science (MBBS) degree. In 2015, the medical program changed to a 4-year graduate-entry doctor of medicine (MD) degree. The UQ medical program averages an annual intake of approximately 498 students. In 2021, 488 students entered the program, comprising 269 domestic Australians, 116 non-Ochsner international students, and 103 UQ-Ochsner Clinical School students. Medical students hailed from 84 birth countries. Most of the 2021 medical student cohort was born in Australia (43%), with the United States (19%), Canada (5%), China (4%), Singapore (4%), and India (3.5%) as the next 5 most common birth countries.

### Rationale for the Program

UQ, already known for its strong international multicultural focus, desired to enrich the domestic student experience with a US partner. The Ochsner partnership was a good fit, providing opportunities for teaching, learning, and research collaborations.

Ochsner sought to establish a strong educational and translational research program to be able to recruit and retain high-quality physicians. After the Hurricane Katrina disaster in 2005, Ochsner grew tremendously and sought a means to train its own medical students, to grow the Ochsner physician workforce, and to contribute to the physician workforce in the United States Gulf South.

### Training Globally to Serve Locally

All UQ-Ochsner Clinical School students complete years 1 and 2 of the medical program in Brisbane. During these preclinical years, students are taught foundational knowledge and skills via a mixture of teaching modalities structured around small-group case-based learning with 10 or fewer students per group. UQ-Ochsner Clinical School students are intermixed in the case-based learning groups with domestic Australian and other international students. These early formative experiences are key to UQ-Ochsner Clinical School student understanding of global perspectives in medicine.

Integral to the partnership's global health perspective is a teaching emphasis on health care in developing countries and health care disparities in disadvantaged populations.^11^ Education in these areas is achieved through global health courses in years 1 and 2 and through a Medicine in Society placement in year 4 that includes experiential learning in developing countries and/or among rural, indigenous, geriatric, or low socioeconomic groups.

For UQ-Ochsner Clinical School students, this global experience included 2 fourth-year optional rotations overseas for which students traveled with Ochsner Clinical School faculty to learn about tropical medicine, social determinants of health, and health care services in a supervised and safe environment. Between 2013 and 2019, up to 6 students each year completed rotations in Haiti, working in clinics run by a Haitian American Ochsner Clinical School faculty member. Security concerns prior to the coronavirus disease 2019 (COVID-19) pandemic caused the Haiti rotation to be suspended after 2019. In 2018 and 2019, up to 8 students per year completed a rotation in Manipal, Karnataka, India. This rotation was suspended with the onset of the COVID-19 pandemic in 2020, with plans to reinstitute it in 2023.

## INITIATIVES AND EXPANSION

### Preclinical Years at The University of Queensland in Brisbane

Clinical preparation in years 1 and 2 combines the study of basic and clinical sciences with training in research, ethics, public health, and clinical skills. Students also complete an 8-week observership within an Australian health care environment between years 1 and 2.

United States Medical Licensing Examination (USMLE) Step 1 preparation for the UQ-Ochsner Clinical School cohort is an important part of their preclinical curriculum. Licensing examinations are not a feature of Australian medical programs, so preparation was not part of the UQ curriculum. The importance of this examination to UQ-Ochsner Clinical School students prompted increasingly comprehensive preparation strategies in years 1 and 2. In 2015 and 2016, all case-based learning tutors received faculty development on the importance of Step 1 for the UQ-Ochsner Clinical School cohort. In 2017, all preclinical UQ course objectives were mapped to USMLE Step 1 objectives, demonstrating coherence of the curriculum with Step 1 concepts. In 2019, a new Step 1 Preparation selective was introduced and required for all UQ-Ochsner Clinical School students. Traditional and other international UQ students could choose to take this selective, or they could choose from 3 other selectives. The Step 1 Preparation course is centered on peer tutorials and resourced with the USMLE UWorld question bank, a well-regarded Step 1 preparation resource. The National Board of Medical Examiners (NBME) Comprehensive Basic Science Self-Assessment is the midsemester examination and the NBME Comprehensive Basic Science Examination is the end-of-semester examination for the Step 1 Preparation course. Students must achieve a passing score on the Comprehensive Basic Science Examination to pass the Step 1 Preparation course, and they must successfully complete the course prior to matriculation into year 3. Starting with the class that matriculated in 2017, all UQ-Ochsner Clinical School students have been required to attempt the USMLE Step 1 before beginning year 3.

### Clinical Years at Ochsner Clinical School in New Orleans

#### Leadership and Staffing

At the origin of the UQ-Ochsner Clinical School partnership in 2009, the chief academic officer (CAO), who had responsibility for research and for undergraduate and graduate medical education, provided leadership of the Ochsner Clinical School. The CAO was the dean of the medical school (known as head of clinical school in Australian parlance). Two assistant deans and 2 administrative personnel managed the Ochsner Clinical School. Over time, as enrollment grew, the leadership team evolved. Currently, the CAO role is separate from the head of school. Three assistant deans (curriculum, student affairs, and admissions), a director of student research, 10 clerkship directors, 5 society heads (explained in the Student Support section), and an administrative team of 11 personnel now support UQ-Ochsner Clinical School activities and deliver the same curriculum provided in Brisbane to the UQ-Ochsner Clinical School student cohort in years 3 and 4.

#### Student Support

In 2014, a new academic center opened in New Orleans, with office space for undergraduate and graduate medical education and research administration, 5 classrooms (capacity of 120 each), a computer laboratory, and a coeducational locker room. These educational spaces were complemented by an 8,300 square foot simulation center that opened in 2017. Renovation in 2020 added space for the recruiting and admissions team.

In January 2017, a society structure was introduced to the Ochsner Clinical School with 5 societies, each named for a founding Ochsner physician and headed by a senior Ochsner Clinical School faculty member. All Ochsner Clinical School students are randomly assigned to a society upon matriculation. Society heads are responsible for mentoring and advising students on their progress through medical school, including taking the USMLE and navigating the US National Resident Matching Program. Prior to the COVID-19 pandemic, society heads visited Brisbane annually to meet with their society members in years 1 and 2 and to interact with faculty on the main UQ campus. A society cup is awarded annually to the society with the most accomplishments in academics, research, and community engagement.

The UQ-Ochsner Clinical School student experience in years 3 and 4 has benefited from incorporating some of the traditions of American medical schools, such as the White Coat Ceremony, that are not part of medical programs in Australia. Ochsner Clinical School introduced the White Coat Ceremony to signify student transition to the next phase in their education, embarking on their clinical training in New Orleans, and as a welcome to the Ochsner Clinical School. Ochsner Clinical School was awarded a chapter of the Gold Humanism Honor Society in 2019 and has initiated 3 classes since then. The Ochsner Clinical School chapter was recognized with an award for excellence in its inaugural year.

#### Research Collaboration

Student research is encouraged through regular research events such as student-led symposiums, seminars, and conferences supported by national and international research partners such as the Australian Academy of Health and Medical Sciences.^12^ International research collaboration has been encouraged through seed funding for translational research projects between UQ and Ochsner Clinical School, MD-PhD scholarships, and fellowship grants for UQ-Ochsner Clinical School graduates to continue their research prior to residency.

Research opportunities in the medical program include an optional research observership between years 1 and 2, a summer research scholarship program, extracurricular research projects, and a higher degree by research (HDR) program (clinician-scientist track) (ie, MD-master's degree or MD-PhD). The Student Research Portal^13^ is the main source of information and guidance for students and research supervisors about medical student research options. The Ochsner Clinical School cohorts have consistently taken advantage of research opportunities. Between 2018 and 2022, Ochsner Clinical School students reported participation in 247 extracurricular research projects, which is 40% of the total reported by the entire UQ medical student cohort. Additionally, Ochsner Clinical School students coauthored 41% of the 195 peer-reviewed journal articles and 57% of the 155 peer-reviewed conference abstracts reported by all UQ medical students during this period.

In 2012, the first Ochsner Clinical School student enrolled in the School of Medicine's clinician-scientist track.^14^ Since then, 17 Ochsner Clinical School students have undertaken an MD-master's degree (n=9) or MD-PhD (n=8), 10 of whom have graduated with both degrees, and the remainder are still progressing through the program. These HDR students benefit from joint supervision and expertise from research faculty at both UQ Brisbane and Ochsner Clinical School New Orleans.

Additional Ochsner Clinical School research support is offered through 6 to 9 postgraduate fellowship grants given annually to graduates during the gap after medical school graduation in December and the start of residency programs in June. These fellowships are based at Ochsner Health, are highly competitive, and include a salary and living expenses.

#### Academic Progression

**Figure 1.**
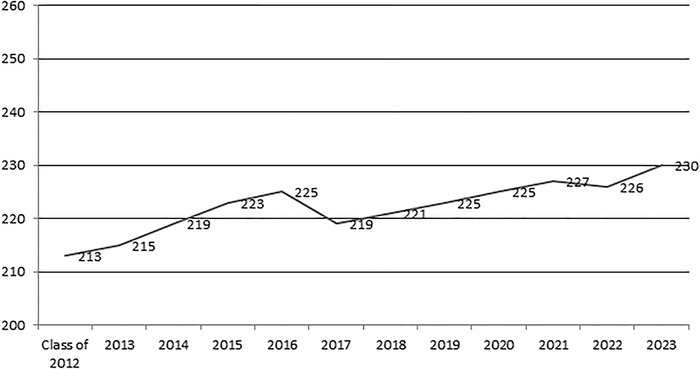
Average passing United States Medical Licensing Examination Step 1 score by year of graduation, Ochsner Clinical School, 2012-2023.

**Figure 2.**
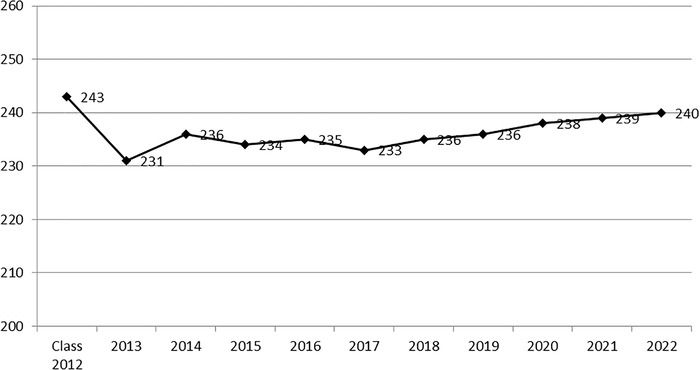
Average passing United States Medical Licensing Examination Step 2 Clinical Knowledge score by year of graduation, Ochsner Clinical School, 2012-2022.

The initial match rates of UQ-Ochsner Clinical School graduates were reported in 2016.^15^ Figures 1 and 2 show the USMLE Step 1 and USMLE Step 2 Clinical Knowledge passing scores by year, respectively.

**Figure 3.**
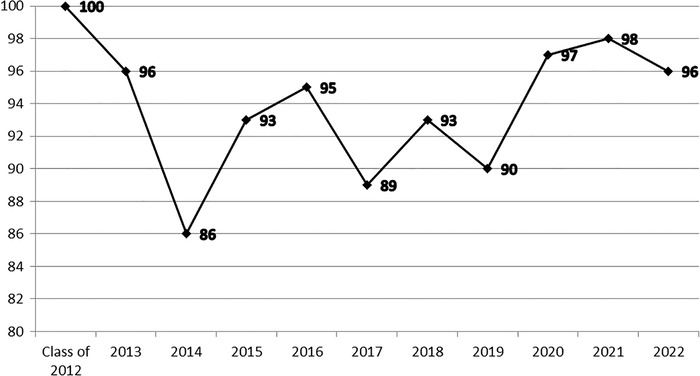
First time match rate of match-eligible graduates by year of graduation, Ochsner Clinical School, 2012-2022.

Table 1 provides another view of student progression. Cumulatively, 78.1% of matriculating students have graduated in 4 years and 87.0% in 5 years. Dropout rates have been low (4.2%) as has dismissal from the program for academic reasons (4.8%), although both of these rates rose during the early part of the COVID-19 pandemic. Figure 3 shows the match rate of match-eligible graduates over time. The overall first-attempt match rate has been 95%. During this period, the average match rate for US MD graduates has been 93% to 94%.

**Table 1.** Matriculation, Graduation, and Attrition Rates by Year of Entry, Ochsner Clinical School, 2009-2019

Entry Year	N	Withdrawn (Personal Reasons)	Dismissed (Academic Reasons)	Transferred to University of Queensland Cohort^a^	4-year Graduates^b^	5-year Graduates	6-year Graduates
2009	16	0	1	4	10	0	1
2010	34	1	1	0	30	2	0
2011	34	2	4	0	26	2	0
2012	80	3	6	0	62	9	0
2013	100	4	1	1	86	6	2
2014	130	6	7	0	103	14	0
2015	120	7	5	0	96	7	5
2016	123	4	5	0	98	12	4
2017	122^c^	3	4	0	92	19	3
2018	110^d^	3	5	0	86	11	1
2019	108^e^	8	8	0	74	5	0

^a^After the class entering in 2009, a policy change strongly discouraged transfers to the traditional Australian University of Queensland cohort.

^b^Students off cycle by 1 rotation are considered in the total number of 4-year graduates.

^c^One student from the class that matriculated in 2017 remains enrolled.

^d^Four students from the class that matriculated in 2018 remain enrolled.

^e^Thirteen students from the class that matriculated in 2019 remain enrolled.

Note: The Australian academic year runs from January through November.

## CHALLENGES MET

### Student Recruitment

At the onset of the program, both UQ and Ochsner Clinical School were concerned about interest and enrollment in the program. A partnership with an external recruiting firm was established, and admission standards were set to ensure projected enrollment numbers were met. Over time, interest has grown along with the reputation of the UQ-Ochsner Clinical School partnership, and academic requirements for admission have been increased. A demographic breakdown of each cohort by entry year is provided in Table 2.

**Table 2.** Age, Sex, Medical College Admission Test (MCAT) Score, and Postgraduate Degrees at Matriculation, Ochsner Clinical School, 2009-2023

Entry Year	N	Average Age at Entry, Years	Percentage Female	Average MCAT Score^a^	Percentage With Master's Degree/PhD
2009	16	26	25	29.4	6
2010	34	27	34	27.9	14
2011	34	26	50	28.6	9
2012	80	25	48	27.6	23
2013	100	25	38	28.5	20
2014	130	25	54	27.8	20
2015	120	26	48	28.8	22
2016	123	27	41	29, 504.7	24
2017	122	26	42	30.1, 503.7	24
2018	110	25	48	30.4, 507.5	21
2019	108	25	55	508	19
2020	118	28	51	509	21
2021	115	27	50	508	25
2022	101	25	50	508	25
2023	90	26	54	510	30

^a^MCAT scoring changed in 2015, so the mean for both formats is shown for 2016 through 2018. Beginning in 2019, all students had 3-digit scores. The minimum MCAT requirement for Ochsner Clinical School entry was increased from 499 in 2016, to 502 with the class entering in 2018, and to 504 with the class entering in 2019.

### Licensing Examinations

As previously discussed, the USMLE Step examinations are not a requirement for licensure in Australia, and the cultural shift to emphasize USMLE preparation in years 1 and 2 was a challenge.

### Accreditation

Two states, California and New York, would not initially allow Ochsner Clinical School graduates to match into residency positions without separate accreditation visits. The Medical Board of California visited the Ochsner Clinical School in March 2014 and granted accreditation in May 2014. The New York State Education Department visited Brisbane in October 2016 and granted accreditation in March 2017.

### International Medical Graduates

Ochsner Clinical School students are considered international medical graduates for the purpose of the US residency match because the school is accredited by the Australian Medical Council. International medical graduates have fewer interview opportunities for some competitive residency programs, and this restriction was a challenge for our early graduates. However, with the achievements of Ochsner Clinical School graduates over time and the development of the partnership's reputation, international medical graduate status has become less of a barrier to match success. As noted in Figure 3, the Ochsner Clinical School match rate is comparable to the US allopathic average, and Ochsner Clinical School graduates have matched in every specialty and in nearly every state.

### Faculty Development

At the onset of the partnership, Ochsner Health physicians who were involved with resident education held academic titles at medical schools in the New Orleans area. Many physicians retained those academic titles and applied for titles at UQ. As of January 2022, 391 Ochsner Health physicians had achieved academic titles at UQ, including 30 full professors.

### COVID 19 Pandemic

The UQ-Ochsner Clinical School partnership was extensively affected by the COVID-19 pandemic, as were medical schools around the globe. Clinical rotations at Ochsner Clinical School began transitioning to a virtual format in mid-March 2020 and were paused for 2 weeks during the height of the initial COVID-19 outbreak in New Orleans. Rotations resumed after this pause, with clinical exposure in lower risk environments and virtual experiences otherwise. Students were strictly prohibited from interacting with patients with active or suspected COVID-19 in all clinical settings. During the pause in clinical rotations, students were offered the opportunity to volunteer for several COVID-19–related research and patient outreach projects, and many students spent many hours volunteering during the early phases of the pandemic. When vaccinations became available, Ochsner Clinical School students were given the same priority as frontline clinicians and staff. Once they were vaccinated, Ochsner Clinical School students were allowed to interact with all patients again.

The Australian approach to the COVID-19 pandemic was different than the US approach. Travel into Australia was prohibited for all but Australian citizens. In 2020, the first-year Ochsner Clinical School class in Brisbane was advised to stay in place through the break between years 1 and 2, and most students followed that advice. Some students elected to return to the United States and could not return to Australia. UQ offered a master of public health degree program for these students at half-price tuition, and 5 of the year 2 students took advantage of this option, which could be completed remotely. The 2021 class spent their entire first year learning remotely and did not enter Australia until they started their second year in 2022. To make up the missed clinical requirements from year 1, all students undertook a mandatory intensive clinical course upon arrival in Brisbane prior to starting year 2. All clinical requirements for year 1 for this cohort were loaded into year 2.

## OBJECTIVES MET

UQ has developed a distinctive educational relationship with a large US health system, and Ochsner Health has developed the medical school relationship that it desired.^16^ A new 10-year agreement began in January 2020 with further enhancements to the program. The overarching aim of the partnership, “train globally to serve locally,” has endured.^17^ Through their training in this partnership, UQ-Ochsner Clinical School graduates bring a unique global outlook to their roles while helping to fill the increasing need for physicians in the United States. The partnership has enhanced the skill sets of domestic and international UQ students, medical educators, and research collaborators.

## CONCLUSION

The UQ-Ochsner Clinical School partnership has been successful for the institutions involved and the students who have graduated. Despite Medical College Admission Test scores slightly lower than typical cut-points for US medical schools, students in the partnership have been successful. By training students in different educational, social, cultural, and medical environments, graduates have a broad perspective on global health and health care delivery.

## Notes

*This article meets the Accreditation Council for Graduate Medical Education and the American Board of Medical Specialties Maintenance of Certification competencies for Medical Knowledge and Systems-Based Practice.*
